# Changes of Differential Urinary Metabolites after High-Intensive Training in Teenage Football Players

**DOI:** 10.1155/2020/2073803

**Published:** 2020-03-18

**Authors:** Ben Cao, Shuojia Liu, Lin Yang, Aiping Chi

**Affiliations:** ^1^School of Sports, Shaanxi Normal University, Xi'an, China; ^2^Shaanxi Youth Sports School, Xi'an, China

## Abstract

**Objective:**

The mechanism underlying the fatigue of football players is closely related to the energy depletion and accumulation of metabolites; the present study tries to explore the metabolic mechanism in teenage football players during exercise-induced fatigue.

**Methods:**

12 teenage football players were subjected to three groups of combined training by using a cycle ergometer, with the subjective Rating of Perceived Exertion (RPE) as a fatigue criterion. The following indicators were measured in each group after training: maximum oxygen uptake (VO_2max_), anaerobic power, and average anaerobic power. Urine samples were collected before and after the training. Gas chromatography-mass spectrometry (GC-MS) was performed for the metabonomics analysis of the samples. The metabolism data was analyzed by using principal component analysis (PCA) and orthogonal partial least squares analysis (OPLS-DA), through the Kyoto Encyclopedia of Genes and Genomes (KEGG) database to confirm the potential differences between metabolites, and the MetPA database was used to analyze the related metabolic pathways.

**Results:**

There was no significant difference between the maximal oxygen uptakes among the three groups. Compared with group 1, the maximum and average anaerobic power in group 3 significantly decreased (*p* < 0.05) at the end of training. GC-MS detected 635 metabolites in the urine samples. Through PCA, OPLS-DA analysis, and KEGG matching, 25 different metabolites (3↑22↓) that met the conditions were finally selected. These different metabolites belonged to 5 metabolic pathways: glycine-serine-threonine metabolism, citrate cycle, tyrosine metabolism, nitrogen metabolism, and glycerophospholipid metabolism.

**Conclusions:**

During the combined exercise of aerobic and anaerobic metabolism, teenage football players show a significant decrease in anaerobic capacity after fatigue. The metabolic mechanism of exercise fatigue was related to disorders in amino acid and energy metabolism.

## 1. Introduction

Exercise-induced fatigue refers to the inability of the body to maintain the predetermined exercise intensity, causing a temporary decline of exercise capacity. But, after appropriate rest and adjustment, the body can return to the original exercise capacity [1]. The improvement of an athlete's training level is the virtuous circle of “fatigue-recovery.” If exercise-induced fatigue cannot well recover, then the athlete will experience overfatigue or extreme fatigue, which can affect exercise ability and health. Therefore, monitoring fatigue is important. Achieving the desired state of fatigue after exercise is needed for scientific training. Additionally, among the commonly used methods of detecting exercise-induced fatigue, recent studies have used metabonomics to examine the metabolic characteristics of human exercise [2–4]. Metabolites can be produced at every level of an organism's cells, organelles, tissues, organs, body fluids, etc. [5] Thus, every physiological activity in the body has to be regulated and influenced by metabolites. Metabolomics is a systematic biological approach to the study of metabolites in the body, focusing on the metabolism of small molecules in biological organisms. Metabonomics research can screen differential markers and examine metabolic pathways and mechanisms. For the metabonomics sample selection, blood and urine are typically used in human tests because of the convenience and noninvasiveness of sampling [6, 7].

Football match is a long-time high-intensity exercise. From the energy metabolism perspective, the energy metabolism of aerobic exercise and anaerobic exercise was combined. Therefore, the mechanism underlying the fatigue of football players is closely related to the energy depletion and accumulation of metabolites. For teenage athletes, their bodies have exuberant metabolism but easily get fatigued. Therefore, the monitoring of fatigue in this age group has practical use in guiding scientific training. The use of a treadmill and a cycle ergometer is appropriate for handling training and establishing a scientific research model. Many studies have used this method as a training model for studying the various exercise intensities of children and adolescents [8–10]. However, there have been several reports on metabolomics studies of adolescents after exercise-induced fatigue. Therefore, in the present study, teenage football players with a certain training background were subjected to a combination of aerobic and anaerobic exercises through a cycle ergometer. After exercise-induced fatigue was reached, urine samples were collected and tested by GC-MS to identify metabolic differences and explore the metabolic mechanism of exercise-induced fatigue.

## 2. Methods

### 2.1. Training Methods

12 male teenage football players from Shaanxi Sports School volunteered to participate in the experiment, and the written informed consents were obtained from their guardians. The tests were performed at 3:00 pm on March 6, 2018. There was no fatigue accumulation on the athletes. 30 min of warming up was permitted. According to the age of the participants, the experimental training model refers to the relevant high-intensity interval training of children and adolescents, and appropriate adjustments were made [8–10].

An alternating exercise of aerobic cycle ergometer (Ergoselect 100 k, Germany) and anaerobic cycle ergometer (MAX-VII, Japan) was used. Each training group included aerobic exercise→rest for 1 min→anaerobic exercise→rest for 3 min, performed in a total of three training times; the aerobic cycle ergometer using the Astrand-Ryhming aerobic exercise mode: 55~60 rpm/min speed for 6 min, and the load was 150 w [11]; and Wingate anaerobic exercise mode: each subject ridded the anaerobic cycle ergometer at fastest speed for 30 s, and each load was set according to their weight according to the direction of Wingate exercise mode [12]. The real-time heart rate was detected by wearing a polar heart rate monitor (RS800sd, Finland). VO_2max_ was counted according to the heart rate at 5 min and 6 min after aerobic exercise, while the maximum anaerobic power, average anaerobic power, and RPE were recorded immediately after training. Fatigue criteria: RPE grades 16 to 19 as the fatigue range, with positive urine protein and urinary gallbladder at 3 h after exercise, and urine protein and urinary gallstones content recovery the next morning to determine exercise-induced fatigue [13–15]. This study was reviewed and approved by Special Committee on Scientific Ethics of Shaanxi Normal University.

### 2.2. Urine Samples

To eliminate interference, such as diet and physical activity, on the day before the experiment, the 12 subjects were provided unified diets and were not permitted to take drugs, tobacco, or alcohol. Dietary standards follow the nutrient intake standard for Chinese young athletes. Mealtime: 7:00-8:00 (breakfast), 12:00-13:00 (lunch), and 18:00-19:00 (dinner). Routine activities and nonintensive exercise were permitted. Urine sampling occurred at two times. The first sampling occurred at 30 min before exercise (preexercise), while the second sampling occurred at 3 h after exercise (postexercise). The urine samples were collected in a 5 mL lidded centrifuge tube, at a sample size of no less than 2 mL. Subsequently, the urine samples were placed in liquid nitrogen cryogenic storage.

### 2.3. GC-MS Assay

A 100 *μ*L aliquot of the urine sample was added to 20 *μ*L of urease and incubated at 37°C for 1 h. Subsequently, 0.35 mL of methanol and 20 *μ*L of L-2-chlorophenylalanine (internal standard) were added, followed by vortexing and centrifugation (13000 rpm, 15 min) at 4°C. Then, 0.39 mL of the supernatant was removed and placed in a 2 mL vial, dried in a vacuum concentrator, mixed with 80 *μ*L of methoxyamine salt reagent, and incubated at 80°C for 30 min; 100 *μ*L of N, O-bis (trimethylsilyl) trifluoroacetamide (BSTFA) was rapidly added to the sample, and the mixture was incubated at 70°C for 120 min. After cooling to room temperature, 10 *μ*L of saturated fatty acid methyl ester standard mixture was added, and the sample was mixed well. After machine test, the Agilent 7890 GC-TOF-MS, equipped with an Agilent DB-5MS capillary column (30 m × 250 *μ*m × 0.25 *μ*m, J & W Scientific, Folsom, CA, USA), was used. The following conditions were implemented for the GC-TOF-MS analysis: injection volume 1 *μ*L; splitless mode; forward sample flow purge flow rate 3 mL/min^−1^; column flow rate 1 mL/min, at a rate of 10°C per minute up to 300°C for 7 minutes; an inlet temperature of 280°C; an ion source temperature of 220°C; and a scanning mode of 50-500 *m*/*z*.

### 2.4. Multidimensional Statistical Analysis

The data of height, weight, and BMI were detected by Shapiro-Wilk test of the SPSS 20.0 to confirm normal distribution, and repeated analysis of general linear model (GLM) was performed on the data of the VO_2max_, maximum anaerobic power, and average anaerobic power. The results were expressed as the means ± standard deviation (^−^*x* ± s) with a significance level of *p* < 0.05.

The Chroma TOF4.3X software (LECO) was used to identify, match, filter out noise, correct baseline, peak alignment, deconvoluted spectra, peak quantify, and quantify the mass spectra of the samples. Internal standard normalization was adopted to reduce the variation in the sample concentrations. Subsequently, multivariate pattern recognition analysis of the normalized data was performed by using SIMCA software (V14, Umetrics AB, Umea, Sweden). Principal component analysis (PCA) and orthogonal partial least squares-discriminant analysis (OPLS-DA) were used to process the complex data obtained between the discrete degrees of the score chart to achieve statistical analysis of the data of each group. All compounds were screened for potential differential metabolites through the Kyoto Encyclopedia of Genes and Genomes (KEGG). The three screening criteria were performed as the previous report: (1) the similarity of the metabolites was greater than 700 when compared to the corresponding peak in KEGG database [16]. (2) The variable importance in the projection (VIP value) of the metabolite was greater than 1. (3) *p* value of the metabolite content was less than 0.05 after Paired-Samples *t*-test. Finally, the selected differential metabolites were entered into the MetPA database (http://www.metaboanalyst.ca) to analyze the influence weights of the corresponding metabolic pathways on screening with a threshold value of more than 0.05, and filtered out after exercise fatigue main metabolic pathways.

## 3. Results

### 3.1. Comparison of Some Physiological Indicators

The basic information of the participants was shown in Table 1. The teenage football players were trained for 3 to 4 years, and their physical qualities were better. The data of height, weight, and BMI were normal distribution after Shapiro-Wilk test, and there were no outliers and extreme values in them.

**Figure 1:**
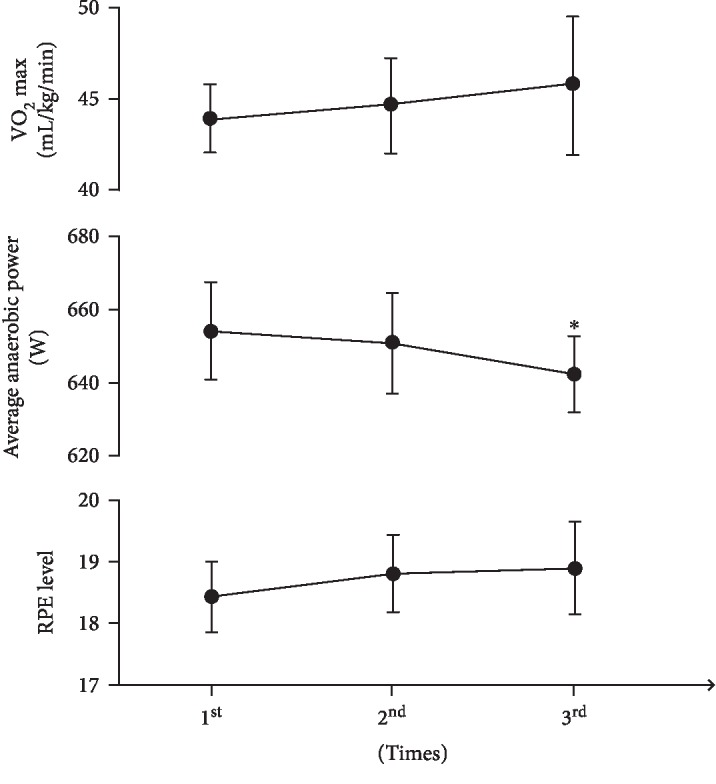
Results of some physiological indices of teenage football players in training (*n* = 12, ^∗^compared with the 1^st^ group; *p* < 0.05).

The changes in the physiological indicators of teenage football players during training were shown in Figure 1; the maximum anaerobic power and average anaerobic power were significantly decreased in group 3 compared with those in group 1 (*p* < 0.05). The changes in other indicators were not obvious. No significant difference was found between the other groups. At the end of each training, the individual RPE reports for each subject ranged from 16 to 19 levels. In addition, urinary protein and urobilinogen showed positive or weak positive results at 3 h after exercise. However, the results of the morning urine test showed that 11 subjects returned to negative results and were determined to have exercise-induced fatigue. One subject did not recover, which may lead to overfatigue. Therefore, in the GC-MS test, the postexercise urine samples for 11 members were tested.

### 3.2. Test Results by GS-MS

**Figure 2:**
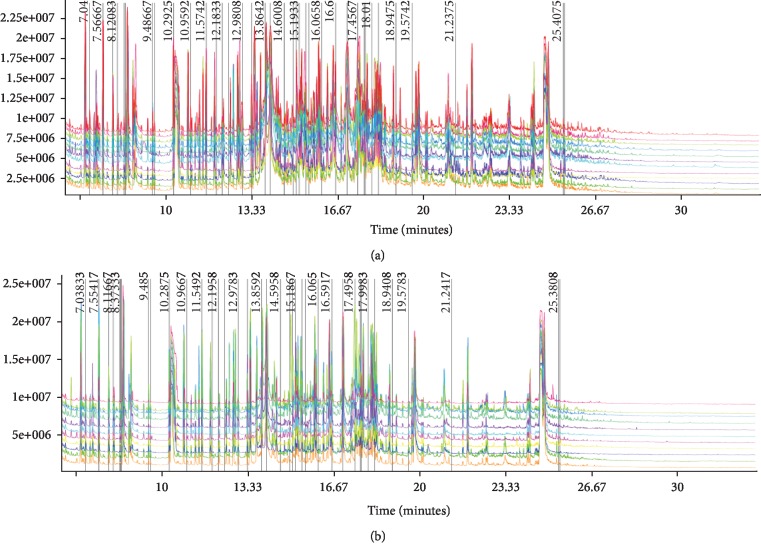
Total ion chromatograms of GC-MS detection. (a) Preexercise. (b) Postexercise.

Urine samples were processed and analyzed by GC-MS to obtain a total ion chromatogram (TIC) as shown in Figure 2. The results showed the total ion chromatogram for the urine samples. A total of 635 peaks were detected before and after training. There is a marked difference between the two groups, indicating that teenage football players had more differences in metabolites before and after training. Therefore, further analysis is necessary. In addition, the chromatograms of total ions in the two groups are neat, and the reproduction of the retention time is good, indicating that the GC-MS instrument used in the present study has high stability and reliability.

### 3.3. PCA and OPLS-DA Analyses of the Urine Metabolites

**Figure 3:**
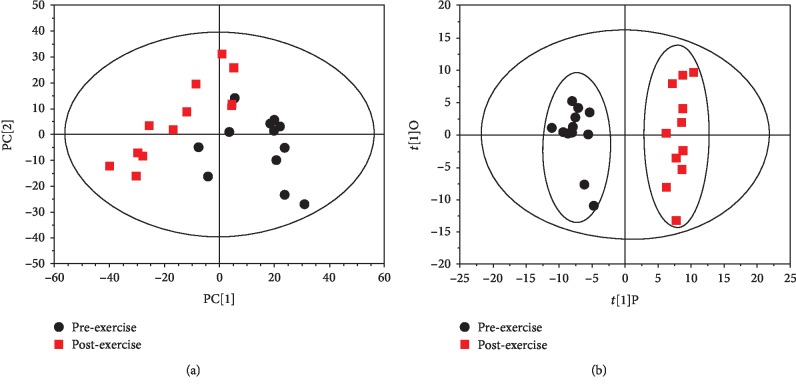
PCA and OPLS-DA analyses of urine metabolites. (a) PCA score. (b) OPLS-DA score.

**Figure 4:**
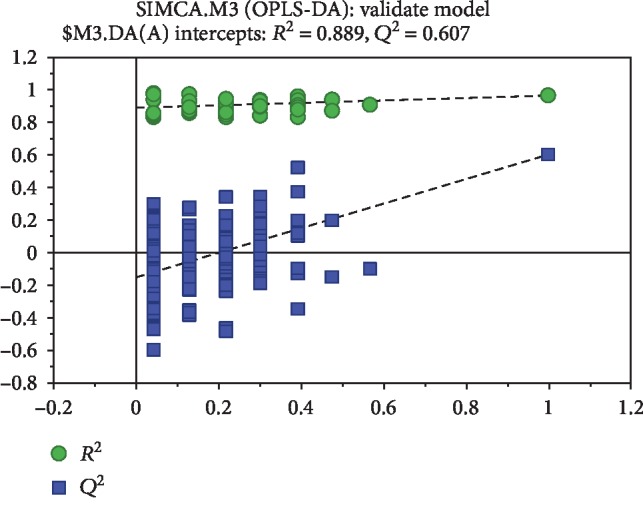
Validation of OPLS-DA model by permutation test.

The 635 peaks obtained above were normalized and filtered. The PCA analysis and OPLS-DA analysis of the normalized data by SIMCA software are shown in Figure 3. The PCA score is an overall presentation of the sample distribution of the raw data. Each point represents a sample, and the coordinates of the sample in the figure are determined by the sample composition, that is, the differences in sample distribution are determined by the differences of composition. The score chart (Figure 3(a)) shows that the scores of preexercise samples are distributed in quadrants I, III, and IV. The scores of postexercise are distributed in quadrants I, II and III. A crossover phenomenon exists between the two samples, and the samples should be further subjected to OPLS-DA analysis to filter out the irrelevant orthogonal signal since the resulting differential metabolites were more reliable. The present study used the VIP (Variable Importance in the Projection) value (threshold > 1) of the first principal component of the OPLS-DA model to determine the differential expression combined with the *p* value of the *t*-test (threshold 0.05) to mark the different metabolites. As shown in the OPLS-DA score chart, the preexercise scores are distributed in quadrants II and III, while the OPLS-DA scores after exercise are distributed in quadrants I and IV (Figure 3(b)). The separation is clearly obvious. The OPLS-DA score can well reflect the similarity of the urine in the group and the sample differences between the groups, indicating a marked difference in the urine metabolites before and after training. Additionally, the model was validated using a permutation test. As shown in Figure 4, *R*^2^ *Y* and *Q*^2^ of the OPLS-DA model were 0.889 and 0.607, respectively, indicating that the model was both reliable and predictive.

### 3.4. Potential Differences in the Screening of Metabolites

By using OPLS-DA analysis, all compounds were screened for differential metabolites through the KEGG database. 25 differential metabolites were identified (VIP > 1, *p* < 0.05, and Similarity > 700). As shown in Table 2, these differential metabolites are mainly involved in amino acid metabolism and energy metabolism. After fatigue, hydroxylamine, citric acid, and sorbitol showed a significant increase (*p* < 0.05 or *p* < 0.01) compared with that before training. These three metabolites are related to nitrogen metabolism, tricarboxylic acid metabolism, and galactose metabolism. The other remaining 22 differential metabolites were significantly decreased after exercise.

### 3.5. Metabolic Pathway Analysis

**Figure 5:**
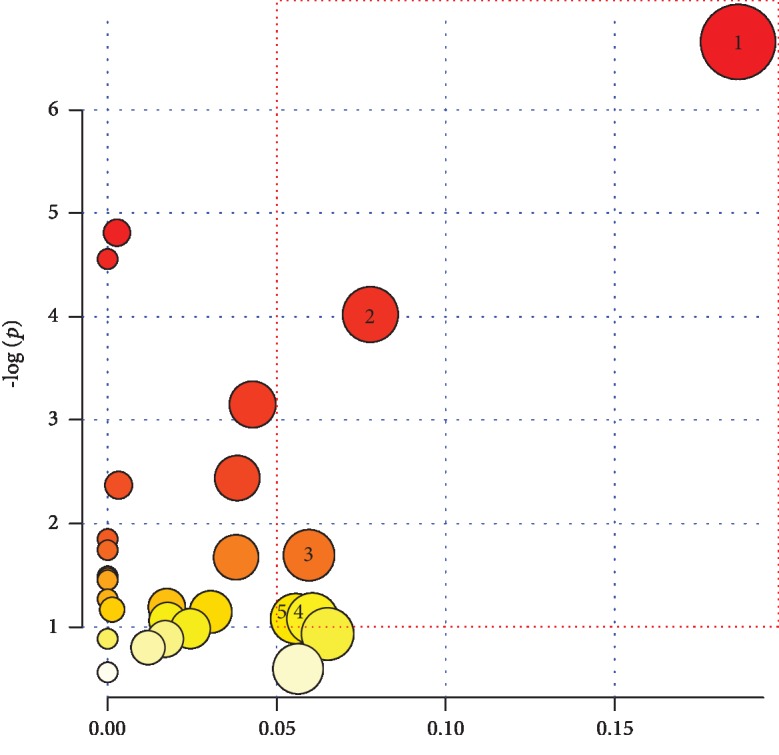
The impact score of the metabolic pathways (1: glycine, serine, and threonine metabolism; 2: citrate cycle; 3: tyrosine metabolism; 4: nitrogen metabolism; 5: glycerophospholipid metabolism).

The above results showed 25 different metabolites involved in many metabolic pathways. In order to identify possible metabolic pathways affected by intensive training, the biomarkers mentioned above were analyzed through MetPA (http://www.metaboanalyst.ca/). Subsequently, their pathway impact values were calculated via a pathway topology analysis. The selected criterion of different metabolites were the impact scores > 0.05 and −log (*p*) > 1 [17, 18]. The selected metabolic pathway is a potential target pathway for exercise-induced fatigue in teenage soccer players. As shown in Table 3 and Figure 5, in accordance with the screening criteria, 5 metabolic pathways were detected: glycine-serine-threonine metabolism, tricarboxylic acid cycle, tyrosine metabolism, nitrogen metabolism, and glycerophosphate metabolism. This finding suggests that the exercise-induced fatigue of teenage football players is due to the disorder among the five metabolic pathways described above.

## 4. Discussion

From an energy metabolism perspective, football is a combination exercise of aerobic metabolism and anaerobic metabolism. Football has high requirements on the aerobic capacity and anaerobic capacity of an athlete. Therefore, it is important to strengthen the comprehensive metabolic ability of the athlete in training. To explore the variation characteristics of differential metabolites and metabolic pathways after exercise-induced fatigue in teenage soccer players, the present study designed a cycle ergometer training model combining aerobic exercise and anaerobic exercise. The results showed that the VO_2max_ of teenage football players remained at a constant level during the three training sessions, but the maximum anaerobic power and average anaerobic power showed a decreasing trend. The third group significantly differs from the first group. The heart rate and RPE reports showed that the athletes had achieved a tired level. According to urinary protein and urobilinogen tests, 11 athletes reached the state of exercise-induced fatigue. Because 1 person experienced extreme fatigue, only 11 samples were used for the urine metabolomics GC-MS detection.

Metabolomics is currently at the forefront of biological detection methods, and many studies use metabolism in sports training, including aerobic exercise [19, 20], anaerobic exercise [21, 22], and physical activity in the general population [23, 24]. From the perspective of the kinetics model, relevant metabolic pathways, HIIT, and changes in bioenergetic metabolic pathways, such as lipid metabolism, protein metabolism, and tricarboxylic acid cycling, are analyzed [25]. However, moderate-intensity continuous training does not show any changes in these metabolic pathways [26]. Therefore, exercise intensity may be the key factor that causes changes in the metabolic pathways of the athletes. The exercise model designed in the present study is a combination of aerobic exercise and anaerobic exercise. This model belongs to high-intensity gap training. After training, the athlete experiences fatigue. The experimental results show that there are 5 metabolic pathways in teenage football players after exercise: glycine-serine-threonine metabolism, tricarboxylic acid metabolism, tyrosine metabolism, nitrogen metabolism, and glycerol phospholipid metabolism. These results can be summarized as three types of metabolism: protein metabolism, tricarboxylic acid cycle metabolism, and lipid metabolism. This result is similar to that of Radom-Aizik et al. [27]

In high-intensity training, sugar and glycogen metabolism is the main energy sources for body movement, but with the extension of exercise time, some variable proteins and amino acids are also involved in energy metabolism to maintain constant exercise intensity [1]. Some studies have found that the urinary levels of histidine and glycine were decreased after exercise in weightlifters, and the levels of histidine and tyrosine in middle- and long-distance runners were also decreased [28]. After 14-18 years of age in power cycling, the contents of glycine-serine-threonine, branched chain amino acids, arginine-proline, glutamic acid-alanine-aspartic acid, etc. were decreased in the urine [27]. The above studies show that different items, different ages, and other factors have different effects on the amino acid metabolism after exercise. These results also showed that in the glycine-serine-threonine metabolic pathway, the four metabolites creatine, L-threonine, creatine, and serine significantly decreased after exercise. In the tyrosine metabolic pathway, amber acid and 4-hydroxyphenylacetic acid were significantly decreased. In the nitrogen metabolism pathway, the hydroxylamine content was significantly increased. These results indicate that amino acid and protein metabolism are increased in athletes during the high-intensity gap training in this experiment. The results of urinary protein and urobilinogen tests and the results of nitrogen metabolism after exercise showed a certain degree of “negative nitrogen” in the athletes, resulting from fatigue after exercise.

The tricarboxylic acid cycle is essential for the catabolism of carbohydrates, fats, and protein. This metabolic pathway not only provides the energy needed for body movement but also provides intermediates for the biosynthesis of many substances [29]. A study conducted by Suzhou University found that the succinate content was decreased and that the citric acid content was increased in athletes after long-distance running, while these parameters did not significantly change in athletes after weight-lifting exercise [28]. The results of the present study showed that succinic acid, a significant metabolite associated with the tricarboxylic acid cycle, was significantly decreased and that citric acid was significantly increased, indicating that exercise-induced fatigue led to the disorder of the tricarboxylic acid cycle in the body, consistent with the results of the above study.

Fatty acid metabolism is the main mechanism of fat metabolism during exercise. Fatty acids are important energy substances in the body. Fatty acid metabolism is closely related to long-term exercise and moderate and low exercise intensity [30]. However, the results of the present study only detected a significant decrease in the content of ethanolamine, a metabolite related to the metabolic pathway of glycerophosphate. Glycerophospholipids are the most abundant types of phospholipids in the body. These compounds constitute biofilms are components of bile and membrane surface-active substances and participate in the recognition and signal transduction of proteins by the cell membrane [31]. A previous study found that acute high-intensity exercise can cause changes in glycerophospholipid metabolism, thus affecting the function of the cell membrane [32]. The experiment showed that disorders of metabolic pathway of glycerol ester are related to the high-intensity training.

## 5. Conclusions

The present study used a cycle ergometer to establish a combined training model of aerobic exercise and anaerobic exercise to ultimately achieve the body fatigue state of teenage football players after exercise. Differential metabolites and related metabolic pathways showed that the metabolic mechanism of exercise-induced fatigue in teenage football players was related to the metabolism of glycine-serine-threonine, tricarboxylic acid cycle, tyrosine metabolism, nitrogen metabolism, and glycerophosphate metabolism. In addition, a variety of the differential metabolites detected here can be used as urinalysis biomarkers for exercise-induced fatigue in teenage football players. The above findings have practical applications in managing the exercise-induced fatigue of football players in this age group and monitoring scientific training.

## Figures and Tables

**Table 1:** Basic information of the exercise participants (*n* = 12).

Age (years)	Training time (years)	Height (m)	Weight (kg)	BMI (kg/m^2^)
14−16	3−4	1.68 ± 0.04	55.31 ± 2.97	19.65 ± 1.01

**Table 2:** Information of the selected differential metabolites after training.

No.	*t*_R_ (min)	Similarity	VIP	KEGG	Metabolites	Molecular formula	*p* value	Fold change*^a^*	Trend*^b^*
1	7.04	901	1.6	C02502	2-Hydroxypyridine	C_5_H_5_NO	9.334*E*-03	1.387	↓^∗∗^
2	7.56	939	1.3	C00160	Glycolic acid	C_2_H_4_O_3_	3.388*E*-02	1.584	↓^∗^
3	8.12	942	1.5	C00192	Hydroxylamine	NH_3_O	6.903*E*-03	0.756	↑^∗∗^
4	8.44	869	1.6	C00213	Sarcosine	C_3_H_7_NO_2_	2.891*E*-03	1.510	↓^∗∗^
5	10.29	867	1.7	C00189	Ethanolamine	C_2_H_7_NO	1.148*E*-03	1.804	↓^∗∗^
6	10.97	822	2.0	C00042	Succinic acid	C_4_H_6_O_4_	7.766*E*-05	3.262	↓^∗∗∗^
7	11.56	907	1.2	C00065	Serine	C_3_H_7_NO_3_	3.582*E*-02	1.575	↓^∗^
8	11.96	886	1.7	C05519	L-Allothreonine	C_4_H_9_NO_3_	1.076*E*-03	2.272	↓^∗∗^
9	12.18	747	1.2	C00489	Glutaric acid	C_5_H_8_O_4_	1.358*E*-04	4.344	↓^∗∗∗^
10	12.98	834	1.0	C00872	Aminomalonic acid	C_3_H_5_NO_4_	1.485*E*-04	3.351	↓^∗∗∗^
11	13.87	772	1.5	C01108	Pyrogallol	C_6_H_6_O_3_	2.651*E*-02	2.296	↓^∗^
12	14.07	797	1.8	C00300	Creatine	C_4_H_9_N_3_O_2_	4.041*E*-03	3.987	↓^∗∗^
13	14.60	892	1.8	C03761	3-Hydroxy-3-methylglutaric acid	C_6_H_10_O_5_	3.848*E*-04	2.108	↓^∗∗∗^
14	14.95	855	1.7	C00156	4-Hydroxybenzoic acid	C_7_H_6_O_3_	3.561*E*-03	2.170	↓^∗∗^
15	15.08	792	1.7	C00642	4-Hydroxyphenylacetic acid	C_8_H_8_O_3_	1.853*E*-03	2.369	↓^∗∗^
16	15.44	871	1.6	C08353	Ribose	C_5_H_10_O_5_	8.630*E*-04	1.790	↓^∗∗∗^
17	15.57	736	1.8	C03139	Guanidinosuccinic acid	C_5_H_9_N_3_O_4_	1.532*E*-04	2.247	↓^∗∗∗^
18	16.07	882	1.8	C03722	Quinolinic acid	C_7_H_5_NO_4_	5.011*E*-04	2.031	↓^∗∗∗^
19	16.60	746	1.6	C11527	4-Hydroxymandelic acid	C_8_H_8_O_4_	4.214*E*-03	1.823	↓^∗∗^
20	17.03	867	1.3	C00158	Citric acid	C_6_H_8_O_7_	4.131*E*-02	0.518	↑^∗^
21	17.70	895	1.2	C00198	Gluconic lactone	C_6_H_10_O_6_	2.088*E*-02	1.383	↓^∗^
22	17.75	910	1.8	C00031	Glucose	C_6_H_12_O_6_	3.691*E*-03	3.907	↓^∗∗^
23	18.01	724	1.3	C00159	Mannose	C_6_H_12_O_6_	1.309*E*-02	1.435	↓^∗^
24	18.26	797	1.6	C00794	Sorbitol	C_6_H_14_O_6_	4.359*E*-02	0.392	↑^∗^
25	21.24	758	1.5	C02470	Xanthurenic acid	C_10_H_7_NO_4_	7.551*E*-06	5.567	↓^∗∗∗^

Fold change*^a^* refers to the ratio of the average metabolite level in postexercise group relative to that in preexercise group.

Trend*^b^* refers to the changed trend of the average metabolite level in postexercise group relative to that in preexercise group. ↑: increase; ↓: decrease; ^∗^*p* < 0.05; ^∗∗^*p* < 0.01; ^∗∗∗^*p* < 0.001.

**Table 3:** Impact scores for metabolic pathways after exercise.

Pathway name	Match status	*p*	−log(*p*)	FDR	Impact	Results^a^
Glycine, serine, and threonine metabolism	4/48	0.001	6.97	0.07	0.187	**√**
Glyoxylate and dicarboxylate metabolism	3/50	0.011	4.49	0.29	0.010	
Citrate cycle (TCA cycle)	2/20	0.015	4.20	0.29	0.078	**√**
Pentose phosphate pathway	2/32	0.036	3.31	0.58	0.043	
Galactose metabolism	2/41	0.057	2.86	0.76	0.002	
Tyrosine metabolism	2/76	0.163	1.81	1.00	0.059	**√**
Arginine and proline metabolism	2/77	0.166	1.79	1.00	0.038	
Methane metabolism	1/34	0.280	1.27	1.00	0.017	
Propanoate metabolism	1/35	0.287	1.24	1.00	0.001	
Ubiquinone and other terpenoid-quinone biosynthesis	1/36	0.294	1.22	1.00	0.030	
Glycerophospholipid metabolism	1/39	0.314	1.15	1.00	0.056	**√**
Nitrogen metabolism	1/39	0.314	1.15	1.00	0.060	**√**
Butanoate metabolism	1/40	0.321	1.13	1.00	0.017	
Nicotinate and nicotinamide metabolism	1/44	0.347	1.05	1.00	0.024	
Lysine degradation	1/47	0.366	1.00	1.00	0.065	
Fructose and mannose metabolism	1/48	0.372	0.98	1.00	0.008	
Starch and sucrose metabolism	1/50	0.384	0.95	1.00	0.017	
Cysteine and methionine metabolism	1/56	0.419	0.86	1.00	0.012	
Aminoacyl-tRNA biosynthesis	1/75	0.519	0.65	1.00	0.056	

^a^Selected criterion was the impact scores > 0.05 and −log (*p*) > 1.

## Data Availability

All data generated or used during the study appear in the submitted article. Some raw data generated or used during the study are available from the corresponding author by request. (List items: Table 2 and Figure 1)
